# A New Cooperative MIMO Scheme Based on SM for Energy-Efficiency Improvement in Wireless Sensor Network

**DOI:** 10.1155/2014/975054

**Published:** 2014-02-20

**Authors:** Yuyang Peng, Jaeho Choi

**Affiliations:** ^1^Department of Electronic Engineering, Chonbuk National University, Jeonju 561-756, Republic of Korea; ^2^CAIIT, Chonbuk National University, Jeonju 561-756, Republic of Korea

## Abstract

Improving the energy efficiency in wireless sensor networks (WSN) has attracted considerable attention nowadays. The multiple-input multiple-output (MIMO) technique has been proved as a good candidate for improving the energy efficiency, but it may not be feasible in WSN which is due to the size limitation of the sensor node. As a solution, the cooperative multiple-input multiple-output (CMIMO) technique overcomes this constraint and shows a dramatically good performance. In this paper, a new CMIMO scheme based on the spatial modulation (SM) technique named CMIMO-SM is proposed for energy-efficiency improvement. We first establish the system model of CMIMO-SM. Based on this model, the transmission approach is introduced graphically. In order to evaluate the performance of the proposed scheme, a detailed analysis in terms of energy consumption per bit of the proposed scheme compared with the conventional CMIMO is presented. Later, under the guide of this new scheme we extend our proposed CMIMO-SM to a multihop clustered WSN for further achieving energy efficiency by finding an optimal hop-length. Equidistant hop as the traditional scheme will be compared in this paper. Results from the simulations and numerical experiments indicate that by the use of the proposed scheme, significant savings in terms of total energy consumption can be achieved. Combining the proposed scheme with monitoring sensor node will provide a good performance in arbitrary deployed WSN such as forest fire detection system.

## 1. Introduction

Wireless sensor network (WSN) has been considered as one of the key techniques in many applications such as environment monitoring, industrial control, and so forth [1]. In a typical WSN, the power supply of each sensor node is from its battery which is energy limited and difficult to charge. Under this situation, minimizing the energy consumption to improve the energy efficiency becomes an alternative way to solve the energy constraint problem. Multiple-input multiple-output (MIMO) has been proved as a core technique to reduce the energy consumption of a wireless network [2]. However, in WSN, wireless sensor node is usually designed by using single transceiver antenna to realize a single-input single-output (SISO) transmission mechanism since the sensor node may not be able to equip using multiple antennas due to the small physical size. Consequently, it is difficult to directly apply MIMO scheme in WSN. Fortunately, the emergence of cooperative MIMO (CMIMO) [3, 4] brings the solution to solve this transmission problem. CMIMO, sometimes referred to as virtual MIMO, can achieve MIMO gains by use of collaboration among the single antennas embedded in each single node. Whereas, in order to realize collaboration, additional circuit and the local data exchange are required which result in the extra energy consumption. Therefore, in order to evaluate the energy consumption performance of CMIMO system, such extra energy also needs to be considered as well as the transmission energy.

Recently, a technique motivated by improving the spectral efficiency named spatial modulation (SM) [5] is proposed. As a working principle, it conveys the incoming information via the spatial antenna index and the MQAM/M-ray phase shift keying symbol in the signal constellation diagram and transmits the modulated symbol through a wireless channel by using a corresponding antenna specified by the antenna index. During the transmission, only one antenna is active while others are sleeping which can effectively avoid the interchannel interference (ICI).

In this paper, in order to take full advantage of these approaches, a new scheme named CMIMO-SM is proposed which involves the joint utilization of cooperative MIMO and SM techniques in WSN for energy-efficiency improvement. We first model the energy consumption of the CMIMO-SM communication and compare the results with the conventional CMIMO. The energy consumption is compared over different transmission distances under the requirement of the same throughput and bit error rate (BER). Three system configurations are considered in both CMIMO-SM and CMIMO system for validating the performance of the proposed scheme, and then the proposed CMIMO-SM scheme is extended to a multihop clustered scenario in which the optimal hop length is derived mathematically by considering the transmitted load. Results from numerical experiments indicate that by use of the proposed scheme, significant performance in terms of energy consumption can be achieved. Also it is demonstrated that the total energy balancing performance of the proposed scheme can be improved.

The remainder of the paper is organized as follows. In Section 2, the related works will be introduced in terms of performance of different CMIMO. In Section 3, the necessary background information including CMIMO and SM are introduced. In Section 4, the proposed scheme and energy model are given. The performance of the proposed scheme and extended one is presented using simulations and numerical experiments in Section 5. Finally, in Section 6 the paper is concluded with a brief summary.

## 2. Related Work 

In [3], for the first time, a CMIMO concept was proposed by Cui et al. for single hop transmission in WSN. It was shown that CMIMO can achieve real MIMO advantages in terms of energy efficient performance if the transmission distance is longer than the critical distance. A vertical Bell labs layered space-time (V-BLAST) based virtual MIMO is proposed in [4], which considers the training overhead requirement. The authors of [6] discussed the efficiency of cooperative transmission under space-time block code-encode (STBC) and the synchronization requirements. In [7], the authors have shown that the number of cooperative nodes at transmission and reception sides is supposed to be selected in order to reduce the energy consumption. The effect of cooperative transmitting area inside the cluster was discussed in [8]. CMIMO in a clustered WSN for energy efficiency was presented in [9]. The routing design to gain the advantages of CMIMO in energy saving is proposed in [10, 11]. In [12], the authors optimized energy consumption per unit transmit distance by selecting the number of cooperative nodes and the transmit energy consumption. The optimization of the cooperative transmission by using single parameter selection of cooperative nodes is introduced in [13, 14]. In [15, 16], the CMIMO with data aggregation technique for energy efficient WSN was presented. In WSN, information collected by the sensors needs to be transmitted to the sink or destination. If the destination is far away, the transmission requires multihop based technique. Finding the optimal hop to benefit CMIMO for achieving energy efficiency was investigated in [17, 18] and multihop hybrid virtual MIMO scheme for WSN was designed in [19]. However, none of these techniques take into account SM which is able to avoid ICI and improve spectral efficiency. To the best of our knowledge, we make the first attempt at changing the transmission way of CMIMO with SM based technique for WSN.

## 3. Background

### 3.1. Cooperative MIMO

The basic idea of CMIMO started with a so called virtual antenna array (VAA) [20], a cooperative scheme wherein one or more antennas were embedded in one sensor node and several sensor nodes cooperate to emulate VAA system to achieve MIMO gains. Compared with real MIMO, CMIMO is more applicable in WSN. It allows small sensor nodes to achieve MIMO function without increasing their physical size. STBC as another important technique in MIMO field makes CMIMO perform well using the diversity approach. Consequently, CMIMO becomes a good candidate to figure out the energy problem in WSN, especially the WSN with energy constraint.

### 3.2. Spatial Modulation

**Figure 1:**
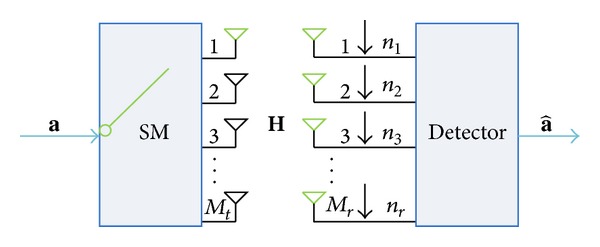
The model of spatial modulation.

Spatial modulation is a practical way for transmitting information by using amplitude/phase modulation and antenna index techniques. This 3 dimensional signal expression approach is able to make SM achieve high spectral efficiency. Furthermore, due to the fact that only one antenna is active during the transmission, the ICI is effectively avoided. The basic working principle is explained using Figure 1. A sequence of bits **a** gets into the SM system which consists of *M*_*t*_ transmitters and *M*_*r*_ receivers. The number of bits that can be transmitted using SM is log_2_(*MM*_*t*_), where *M* = 2^*b*^ and *b* is constellation size. We can write this incoming bits **a** using a vector **x** = [*x*_1_ *x*_2_ ⋯ *x*_*Mt*_]^*T*^, where a power constraint of unity is assumed. According to the working principle of SM, only one antenna is active during the transmission which results in a single nonzero element in **x**. The signal is transmitted from the corresponding antenna over the *M*_*r*_ × *M*_*t*_ MIMO channel **H** whose elements are assumed to be independent identically distributed (i.i.d.) complex Gaussian random variables with zero mean and unit variance, and *M*_*r*_ dimensional noise vector **n** are assumed to be i.i.d. complex Gaussian random variables with zero mean and variance of *N*_0_. Then the received signal can be written as **y** = **Η** **x** + **n** and the estimated symbol can be extracted by using optimal maximum likelihood (ML) detector. Mathematically, the estimated symbol can be expressed by $\hat{x}\;=\arg\;\min_{X\in \Lambda }{||y-Hx||}_{F}^{2}$, where $\hat{x}$ indicates the estimated symbol vector, Λ is the set of all possible transmit symbols, and ||·||_*F*_ denotes the Frobenius norm of the vector.

## 4. System Modeling 

### 4.1. System Model

**Figure 2:**
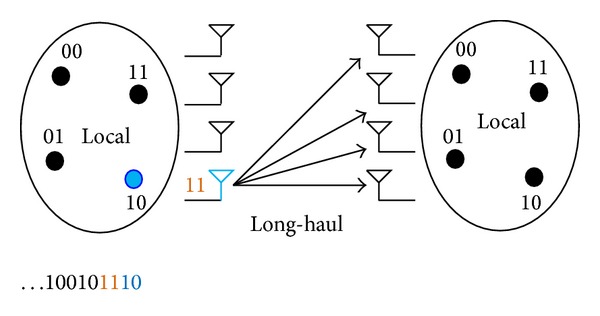
The structure of CMIMO-SM.

Let us consider a wireless sensor network in which each sensor node is equipped with a single antenna for data transmission. Typically, local sensor nodes inside the cluster collect the information and transmit it to the next relay cluster if the destination is far away. As we discussed in the last section, CMIMO can make single node with single antenna achieve MIMO performance and SM can provide high spectral efficiency and avoid ICI. Thus, if we allow CMIMO and SM techniques to be used among multiple nodes, the joint performance will be obtained. Such scenario is shown in Figure 2.

**Figure 3:**
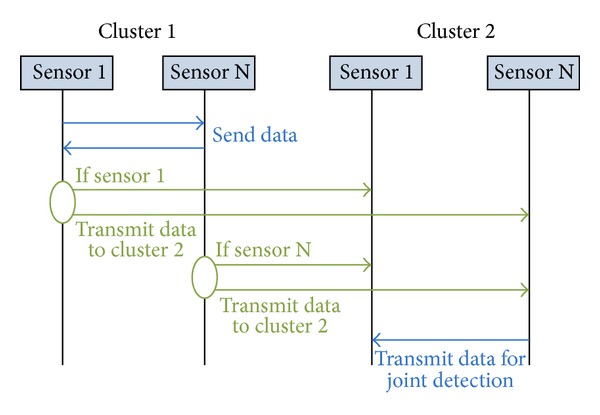
The communication process of CMIMO-SM.

In our proposed model, each sensor node inside the cluster has a preassigned index using binary numbers to represent. In order to realize the MIMO function for saving energy, the local data exchange is necessary. The data flow inside the cluster is defined as local transmission while the data delivering between two clusters is defined as long-haul transmission. Suppose that *M*_*t*_ nodes on the transmitting side while *M*_*r*_ nodes on the receiving side. On the transmitting side, each sensor node broadcasts its information to all the other nodes inside the cluster using different time slots as the first stage. Once each node receives all the other information bits, the data sequence is ready to be transmitted through the MIMO channel. For each time instant, the data sequence is composed by the MQAM/MPSK modulated symbol part and antenna represented part. Only the MQAM/MPSK modulated symbols are transmitted and the symbol represented by corresponding antenna as the hidden information will be detected at receiver. On the receiver side, one destination node and *M*_*r*_ − 1 nodes join the cooperative reception. The whole process of CMIMO-SM can be explained by using Figure 3.

Example: consider a CMIMO-SM based WSN with 4 sensor nodes on the transmitter and each sensor node has an index which is similar to the instance in Figure 2. It is assumed that 1110 is the data sequence to transmit after local data flow and modulation is 4QAM. According to our proposed approach, only bits 11 will be modulated using 4QAM and transmitted via the corresponding antenna 10 while the bits 10 as the antenna index will be detected at the receiver.

### 4.2. Energy Consumption Model

In order to evaluate the energy performance of the proposed scheme, the energy model is first discussed. From [3] it is known that the total average power consumption of a normal communication system can be categorized into two main components: the power consumption of all the power amplifiers *P*_PA_ and the power consumption of all the circuit blocks *P*_*c*_. *P*_PA_ depends on the output transmission power *P*_out_ and has a linear relation:

(1) $$\begin{array}{c} P_{\text{PA}}=\left(1+\alpha \right)P_{\text{out}}, \end{array}$$

where **α** equals to *ξ*/*η* − 1 with *ξ* being the peak to average ratio (PAR) and **η** being the drain efficiency of the RF power amplifiers. For MQAM, *ξ* = 3(*M* ^1/2^−1)/(*M* ^1/2^+1). *P*_out_ can be calculated as below if the channel is a square-law path loss [21]:

(2) $$\begin{array}{c} P_{\text{out}}={\overset{-}{E}}_{b}R_{b}\times \frac{{\left(4\pi d\right)}^{2}}{G_{t}G_{r}\lambda^{2}}M_{l}N_{f}, \end{array}$$

where ${\overset{-}{E}}_{b}$ is the average energy per bit required for a given bit error rate (BER) at receiver, *d* is the transmission distance, *G*_*t*_ and *G*_*r*_ are the transmitter and receiver antenna gains, respectively, **λ** is the carrier wavelength, *M*_*l*_ is the link margin compensating the hardware process variations and other additive interference or background noise, and *N*_*f*_ is the receiver noise figure. It should be noted that *N*_*f*_ is given by *N*_*f*_ = *N*_*r*_/*N*_0_, where *N*_*r*_ is the power spectral density (PSD) of the total effective noise at the receiver input and *N*_0_ is the single-sided thermal noise PSD at room temperature with a value *N*_0_= −171 dBm/Hz. In (2), depending on the number of transmit and receive antennas, ${\overset{-}{E}}_{b}$ can be calculated using the SNR value and the PSD of the thermal noise *N*_0_ for the BER requirement.

The total circuit power consumption for an *M*_*t*_ transmit and *M*_*r*_ receive antennas system is given by

(3) pc≈Mt(PDAC+Pmix+Pfilt)+2Psyn+Mr(PLNA+Pmix+PIFA+Pfilr+PADC),

where *P*_DAC_, *P*_mix_, *P*_LNA_, *P*_IFA_, *P*_filt_, *P*_filr_, *P*_ADC_, and *P*_syn_ are the power consumption values for the DAC, the mixer, the low noise amplifier (LNA), the intermediate frequency amplifier (IFA), the active filters at the transmitter side, the active filters at receiver side, the ADC, and the frequency synthesizer, respectively. The values of *P*_DAC_, *P*_ADC_, and *P*_IFA_ can be calculated using the mode introduced in [22].

The total energy consumption per bit can be expressed as

(4) $$\begin{array}{c} E_{\text{bt}}=\frac{P_{\text{PA}}+P_{c}}{R_{b}}. \end{array}$$

According to (1) and (2), the total energy consumption per bit can be rewritten as

(5) $$\begin{array}{c} E_{\text{bt}}=\left(1+\alpha \right){\overset{-}{E}}_{b}\times \frac{{\left(4\pi d\right)}^{2}}{G_{t}G_{r}\lambda^{2}}M_{l}N_{f}+\frac{P_{c}}{R_{b}}. \end{array}$$

According to the transmission process described in Section 4.1, the energy consumption per bit of the proposed scheme *E*_bt,csm_ consists of two components: energy consumption in the local phase *E*_*l*_ and energy consumption in the long-haul phase *E*_lh_, that is,

(6) $$\begin{array}{c} E_{\text{bt},\text{csm}}=E_{l}+E_{\text{lh}}. \end{array}$$

For the local energy consumption, there are two communication phases: (1) energy consumption of data exchange inside cluster in transmitter side *E*_*i*_^*t*^; (2) energy consumption of data collection for joint detection inside cluster in receiver side *E*_*j*_^*r*^. Note that for the local phase communication, cooperative transmission is not used and *M*_*t*_ = *M*_*r*_ = 1, namely SISO communication. For the long-haul energy consumption, CMIMO-SM is utilized and note that the transmit antenna number *M*_*t*_ is always equal to one in the circuit power consumption, since only one antenna is active during each time instant. Assume that each sensor node has *N*_*i*_ bits to transmit; and then the energy consumption per bit for *E*_*l*_ is given by

(7) $$\begin{array}{c} E_{l}=\frac{\sum_{i=1}^{M_{t}}N_{i}E_{i}^{t}+\sum_{j=1}^{M_{r}-1}E_{j}^{r}\sum_{i=1}^{M_{t}}N_{i}}{\sum_{i=1}^{M_{t}}N_{i}}. \end{array}$$

After adding *E*_lh_ to *E*_*l*_, the energy consumption per bit can be expressed as

(8) $$\begin{array}{c} E_{\text{bt},\text{csm}}=\frac{\sum_{i=1}^{M_{t}}N_{i}E_{i}^{t}+E_{\text{lh}}\sum_{i=1}^{M_{t}}N_{i}+\sum_{j=1}^{M_{r}-1}E_{j}^{r}\sum_{i=1}^{M_{t}}N_{i}}{\sum_{i=1}^{M_{t}}N_{i}}, \end{array}$$

where *E*_*i*_^*t*^, *E*_*j*_^*r*^, and *E*_lh_ can be calculated according to (5).

In this paper, for a realistic case we take into account the detection energy as well. The detection energy can be calculated by using operation complexity in terms of complex multiplication and addition. Processing ability of the multiplications and additions depends on the CPU speed and can be different for different devices. We refer to the TelosB mote [23] as an instance. It requires 8 cycles and 4 cycles for dealing with multiplication and addition, respectively, and *E*_*J*_ Joule per cycle [24]. When SM applies ML at the receiver, 2*M*_*t*_ *M*_*r*_ + (*M*_*t*_ + 1)*M* complex multiplications and *M*_*t*_(*M*_*r*_ − 1) complex additions are needed for one symbol detection [25]. So the energy consumption per bit for detection can be calculated as

(9) $$\begin{array}{c} E_{d}=\frac{E_{J}\times \left\{8\left[2M_{t}M_{r}+\left(M_{t}+1\right)M\right]+4\left[M_{t}\left(M_{r}-1\right)\right]\right\}}{\text{lo}{\text{g}}_{2}\left(M_{t}M\right)}. \end{array}$$

And then the energy consumption per bit for CMIMO-SM approach is given by

(10) $$\begin{array}{c} E_{\text{bt},\text{csm}}=\frac{\sum_{i=1}^{M_{t}}N_{i}E_{i}^{t}+E_{\text{lh}}\sum_{i=1}^{M_{t}}N_{i}+\sum_{j=1}^{M_{r}-1}E_{j}^{r}\sum_{i=1}^{M_{t}}N_{i}}{\sum_{i=1}^{M_{t}}N_{i}}+E_{d}. \end{array}$$

For energy consumption evaluation and comparison, the reference CMIMO introduced in [3] is used in this paper. For the case of CMIMO, energy consumption per bit in the local phase is the same as that in the proposed scheme. For the long-haul phase, Alamouti approach [26] is utilized and when Alamouti applies ML at the receiver, 0.5*M*_*r*_ complex multiplications and 2*M*_*r*_ − 1 complex additions are needed for one symbol detection [5]. The same calculating way as CMIMO-SM, the energy consumption per bit for detection is given as

(11) $$\begin{array}{c} E_{\text{dc}}=\frac{E_{J}\times \left[8\left(0.5M_{r}\right)+4\left(2M_{r}-1\right)\right]}{\text{lo}{\text{g}}_{2}M}. \end{array}$$

The total energy consumption per bit for CMIMO is given as

(12) $$\begin{array}{c} E_{\text{bt},\text{c}}=\frac{\sum_{i=1}^{M_{t}}N_{i}E_{i}^{\text{tc}}+E_{\text{lhc}}\sum_{i=1}^{M_{t}}N_{i}+\sum_{j=1}^{M_{r}-1}E_{j}^{\text{rc}}\sum_{i=1}^{M_{t}}N_{i}}{\sum_{i=1}^{M_{t}}N_{i}}+E_{\text{dc}}, \end{array}$$

where *E*_*i*_^tc^and *E*_*j*_^rc^ represent the local transmission energy cost per bit for cooperation on the transmitter side and joint detection on the receiver side, respectively. The energy consumption per bit in long-haul transmission by using the Alamouti approach is denoted as *E*_lhc_. *E*_*i*_^tc^, *E*_*j*_^rc^, and *E*_lhc_ can be calculated according to (5).

## 5. Simulations and Numerical Experiments 

### 5.1. For Long-Haul Communication

**Figure 4:**
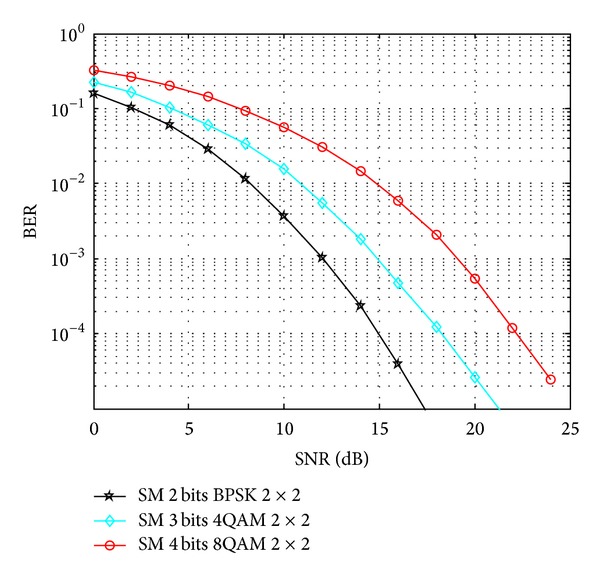
The received SNR versus BER in SM with 3 different modulations.

**Figure 5:**
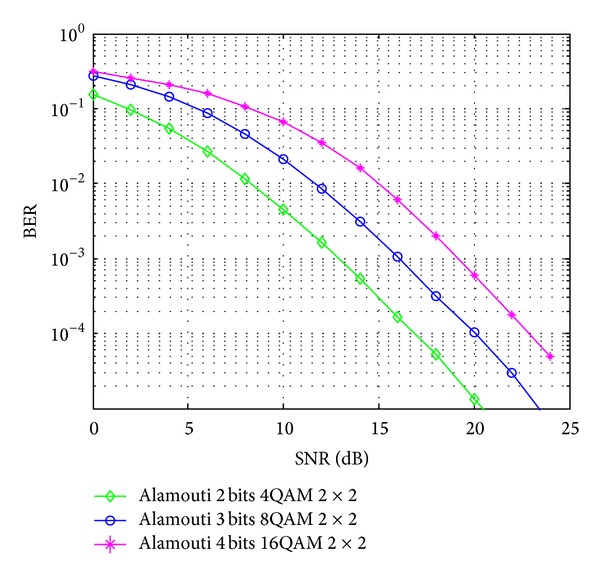
The received SNR versus BER in Alamouti with 3 different modulations.

In our simulation, Monte Carlo simulations are carried out to find ${\overset{-}{E}}_{b}$. Specifically, ten thousand randomly generated channel samples are taken and averaged to find the desired BER and then invert to get the required value of ${\overset{-}{E}}_{b}$. Assume that the rate of 2 bits/s/Hz, 3 bits/s/Hz, and 4 bits/s/Hz can be supported. Figures 4 and 5 represent the BER performance under different SNR values of SM and Alamouti, respectively.

${\overset{-}{E}}_{b}$ is calculated using the SNR value plotted in Figures 4 and 5 and the PSD of the thermal noise *N*_0_ for the BER requirement 10^−3^. The configurations of modulation and **α** of CMIMO-SM and CMIMO in long-haul stage are tabulated in Tables 1 and 2, respectively. Total circuit power consumption *P*_*c*_ is calculated using (3) and listed in Table 3.

### 5.2. For Local Communication

In the local phase, both CMIMO-SM and CMIMO utilize BPSK as the modulation scheme and SISO as transmission approach. The same approach introduced in Section 5.1 is used to simulate and calculate the parameters which are listed in Table 4.

In this simulation, the following values have been used: *B *= 10 kHz, *f*_*c*_ = 2.5 GHz, *P*_mix_ = 30.3 mW, *P*_filt_ = 2.5 mW, *P*_filr_ = 2.5 mW, *P*_LNA_ = 20 mW, *P*_synth_ = 50 mW, *M*_*l*_ = 40 dB, *N*_*f*_ = 10 dB, *G*_*t*_ *G*_*r*_ = 5 dBi, and *η* = 0.35, *E*_*J*_ = 1.215 nJ, *N*_*i*_ = 20 kb. For the fair comparison, we give the same setup *M*_*t*_ = *M*_*r*_ = 2 as in [3].

### 5.3. Energy Consumption Comparison

**Figure 6:**
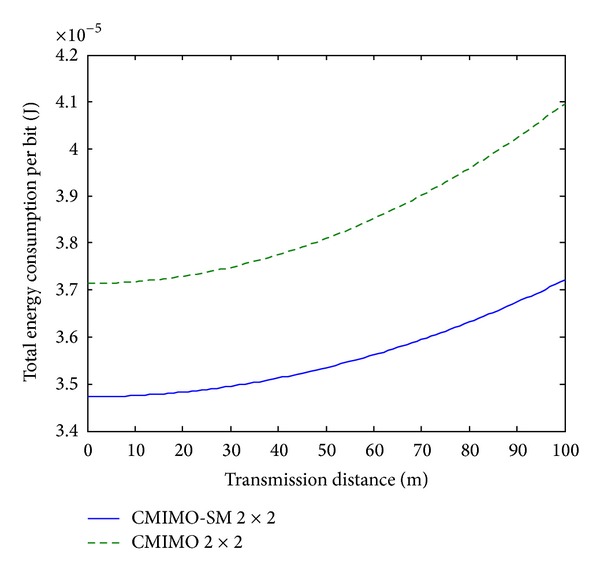
Total energy consumption per bit over *d*, 2 bits transmission.

**Figure 7:**
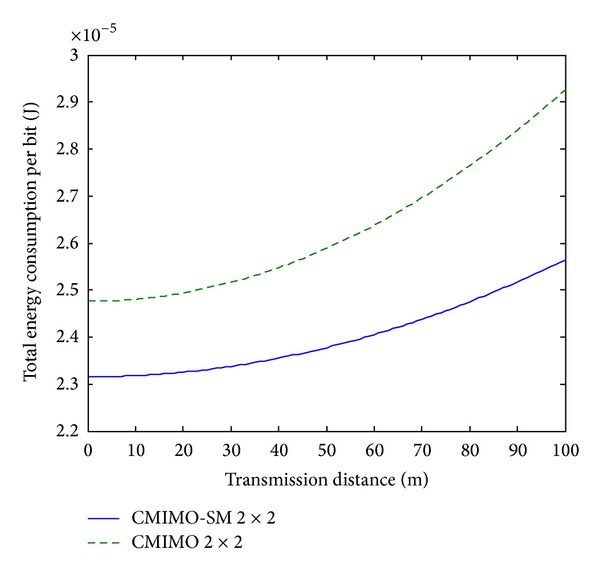
Total energy consumption per bit over *d*, 3 bits transmission.

**Figure 8:**
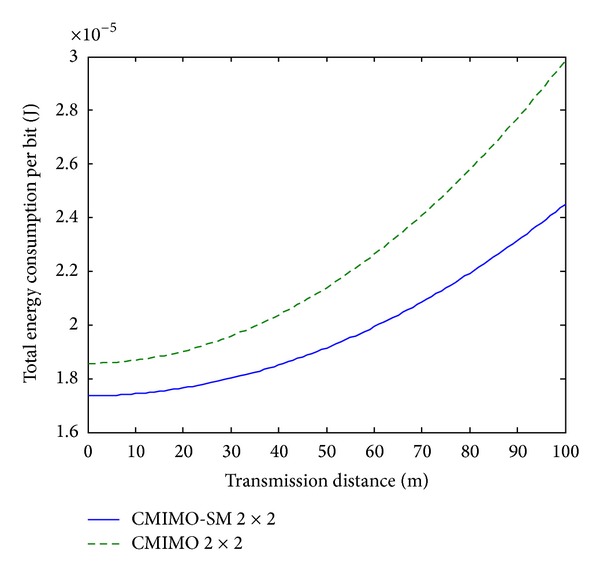
Total energy consumption per bit over *d*, 4 bits transmission.

Since the analytical and simulation results are too complicated to offer obvious information, it is expected that some comparisons can be given from our results. It is assumed that the average distance between two adjacent nodes inside the cluster is about 2 meters. The energy consumption per bit comparisons for 2 bits, 3 bits, and 4 bits systems between CMIMO-SM and CMIMO are presented in Figures 6, 7, and 8, respectively.

From above plots, we see that the proposed CMIMO-SM beats CMIMO due to the advanced transmission scheme. Also, for both systems, as the transmission distance increases, energy consumption per bit increases. Moreover, for both systems, the energy consumption per bit decreases as the transmission rate increases from 2 bits/s/Hz to 4 bits/s/Hz. This can be explained by the reason that circuit power working in a shorter time will bring lower energy.

### 5.4. Energy Consumption in Multihop Scenario

**Figure 9:**
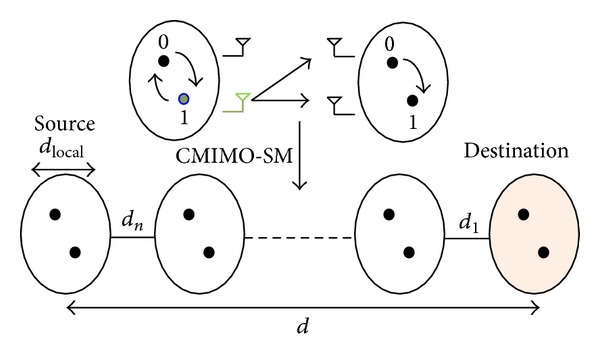
The structure of multihop CMIMO-SM.

Now a multihop WSN composed of *n* clusters and a destination is considered and shown in Figure 9. The proposed CMIMO-SM scheme is applied to this multihop WSN for saving energy. Inside the cluster, the longest distance amongst the nodes is defined as *d*_local_. The long-haul distance between the nearest nodes of different clusters is defined as *d*_*i*_  (*i* = 1,2,…*n*) which is assumed to be much larger than *d*_local_. Let *d*_*i*_ represent the optimal transmission distance and then, for a transmission distance *d*_*i*_, energy consumption per bit of long-haul is given by

(13) $$\begin{array}{c} E_{\text{bt}}^{\left(i\right)}=\left(1+\alpha \right){\overset{-}{E}}_{b}\times \frac{{\left(4\pi d_{i}\right)}^{2}}{G_{t}G_{r}\lambda^{2}}M_{l}N_{f}+\frac{P_{c}}{R_{b}}. \end{array}$$

It will be assumed that two sensor nodes in one cluster are used in order to reduce the complexity of the calculation. However, it is important to note that the calculation can in principle be extended to any cluster size. For all nodes transmitting scenario, the total energy consumption can be expressed as

(14) Etotal=∑i=1n(∑i=1MtNiEit+∑j=1Mr−1Ejr∑i=1MtNi+∑i=1MtNiEd)+2∑i=1n(n+1−i)×[(1+α)E−b×(4π)2×(di+dlocal)2GtGrλ2MlNf+PcRb]Ni.

In order to minimize the total energy consumption, the optimal transmission distance *d*_*i*_ from the source to the destination needs to be derived. First, through observation of the transmission distance in the proposed model, the constraint ∑_*i*=1_^*n*^ *d*_*i*_ = *d* − *nd*_local_ is obtained; and then the optimization problem can be formulated as

(15) min⁡di,  i=1,2,…n  2∑i=1n(n+1−i)×[(1+α)E−b×(4π)2×(di+dlocal)2GtGrλ2MlNf+PcRb]Nis.t∑i=1ndi+ndlocal=ddi>0,  i=1,2,…,nn  is  a  positive  integer.

**Proposition 1**

Under the all nodes transmission case, given the total distance between source and destination *d*, the optimal transmission distance *d*_*i*_ is dependent on *d* and decreases as the cluster approaches to the destination.

**Proof**

See the appendix.

Therefore, setting hop distances using ((A.4)) for all hop-lengths is sufficient for achieving the minimum energy consumption.

**Figure 10:**
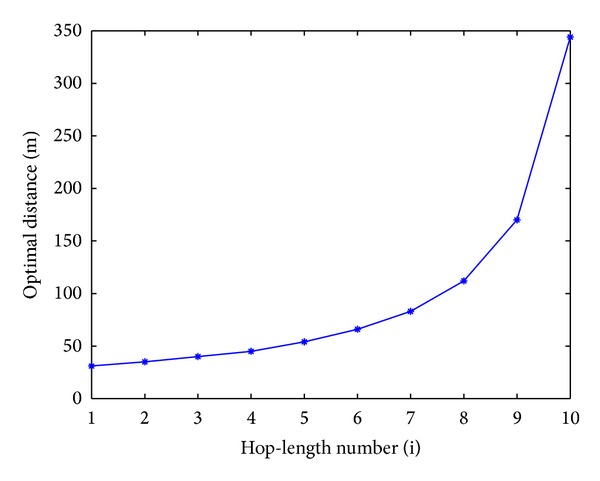
Optimal distances versus hop-length numbers.

The numerical results of the optimization and multihop based CMIMO-SM are presented in Figure 10. As an example of numerical results, suppose that the source and destination are *d* meters apart and *n* − 1 clusters act as relays to forward the information. Assume that *n* is chosen to be 10, *d* and *d*_local_ are set to be 1000 meters and 2 meters, respectively. The optimal transmission distances are obtained using ((A.4)) and plotted in Figure 10. It can be observed that for each hop-length, we can find a best transmission value and also it can be seen that as the distance to the destination increases, the hop-length also increases. The reduction of energy consumption can be further obtained using this approach instead of the equidistant approach.

**Figure 11:**
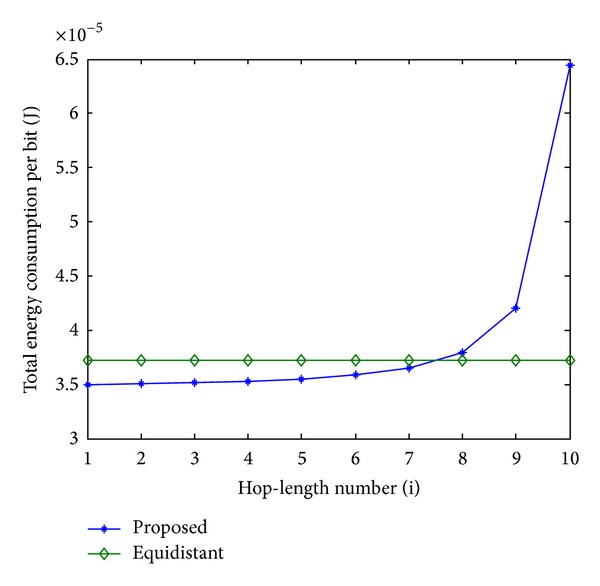
Total energy consumption per bit for each cluster, 2 bits transmission.

**Figure 12:**
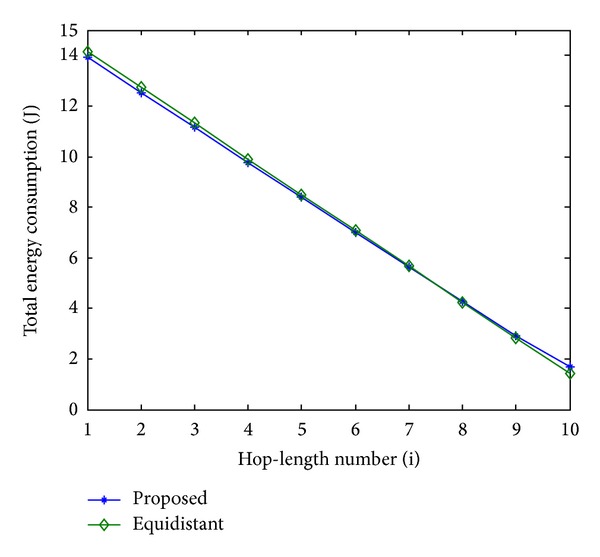
Total energy consumption for each cluster, 2 bits transmission.

**Figure 13:**
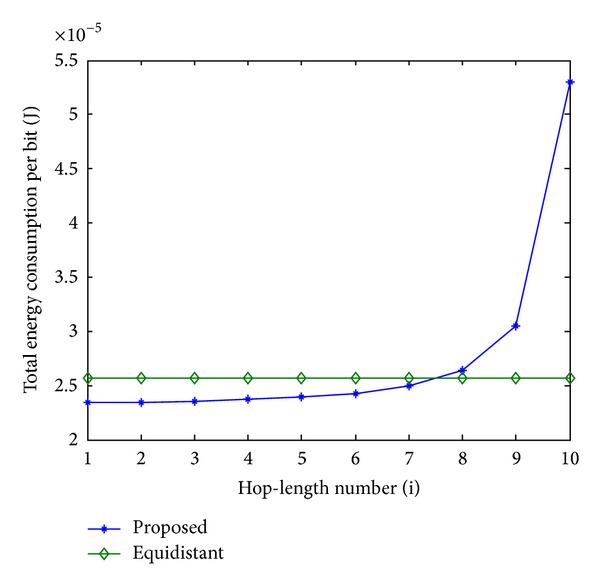
Total energy consumption per bit for each cluster, 3 bits transmission.

**Figure 14:**
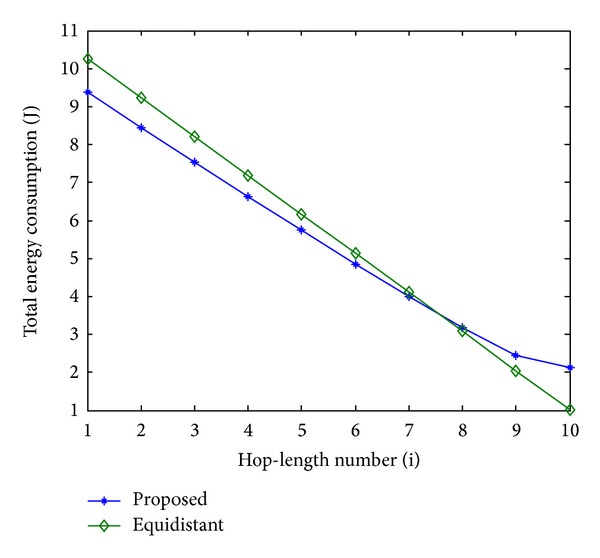
Total energy consumption for each cluster, 3 bits transmission.

**Figure 15:**
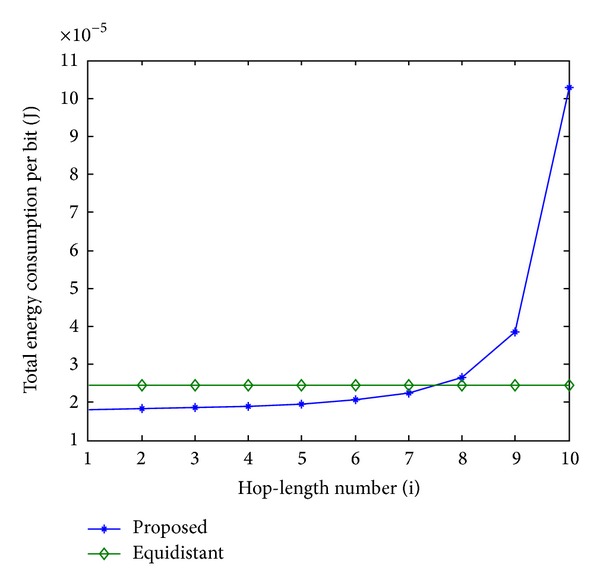
Total energy consumption per bit for each cluster, 4 bits transmission.

**Figure 16:**
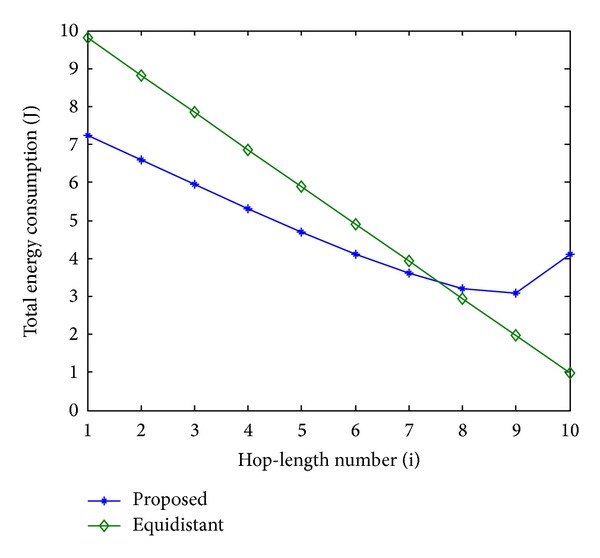
Total energy consumption for each cluster, 4 bits transmission.

In order to evaluate the performance of the proposed scheme, an equidistant scheme as a reference is compared with the proposed scheme in terms of energy consumption. In Figures 11, 13, and 15, the total energy consumption per bit versus hop-length number is plotted as a calculation aim for the proposed scheme and reference scheme in terms of three different system configurations. It can be seen that the majority of clusters in the proposed scheme have less total energy consumption per bit when compared with the equidistant scheme because transmission distances within these hops are relatively short for the proposed scheme, while the opposite reason can be explained to the minority of the clusters. By using this approach, the total energy consumption is effectively reduced and balanced. After calculating total energy consumption of each cluster, the results in Figures 12, 14, and 16 show that the proposed scheme offers a good energy balance property compared to the equidistant scheme for a given transmission distance.

## 6. Conclusions

A new cooperative transmission scheme CMIMO-SM has been proposed in this paper. The system model was designed and the energy consumption was analyzed in a cluster to cluster communication environment under three different system configuration cases. The superiority of using this scheme for minimization of total energy consumption is validated by simulations and numerical experiments. Results demonstrated that the proposed scheme always provides energy saving compared with when using the conventional one. Later, we integrated the CMIMO-SM into a multihop scenario to further optimize the total energy consumption. The results indicated that optimal hop scheme can reduce and balance energy consumption significantly, and thus the feasibility of using CMIMO-SM in optimal multihop scheme to boost the joint performance was proved. Therefore, it is concluded that the proposed scheme can be used as a guideline to design energy efficient communications in arbitrary deployed WSN for the reduction of energy consumption and extension of the lifetime.

## Figures and Tables

**Table 1:** Modulation configurations for 2 bits/s/Hz, 3 bits/s/Hz, and 4 bits/s/Hz transmissions using CMIMO-SM and CMIMO.

	2 bits/s/Hz	3 bits/s/Hz	4 bits/s/Hz
CMIMO-SM	BPSK	4QAM	8QAM
CMIMO	4QAM	8QAM	16QAM

**Table 2:** *α* configurations for 2 bits/s/Hz, 3 bits/s/Hz, and 4 bits/s/Hz transmissions using CMIMO-SM and CMIMO.

	2 bits/s/Hz	3 bits/s/Hz	4 bits/s/Hz
CMIMO-SM	0.4706	1.8571	3.0937
CMIMO	1.8571	3.0937	4.1429

**Table 3:** Circuit power consumption *P*_*c*_ for 2 bits/s/Hz, 3 bits/s/Hz, and 4 bits/s/Hz transmissions using CMIMO-SM and CMIMO.

	2 bits/s/Hz	3 bits/s/Hz	4 bits/s/Hz
CMIMO-SM		0.2732 W	
CMIMO		0.3214 W	

**Table 4:** *α*, and *P*_*c*_ values for local communication using CMIMO-SM and CMIMO.

	*α*	*P*_*c*_
CMIMO-SM/CMIMO	0.4706	0.2107 W

## Appendix

### Proof of Proposition

To solve the problem in (15) under the constraint ∑_*i*=1_^*n*^ *d*_*i*_ = *d* − *nd*_local_, we set

(A.1) L=2∑i=1n(n+1−i)×[(1+α)E−b×(4π)2×(di+dlocal)2GtGrλ2MlNf+PcRb]Ni+w(d−ndlocal−∑i=1ndi),

where *w* is a Langrage multiplier. According to the method of Langrage multiplier, we have

(A.2) $$\begin{array}{c} \frac{\partial L}{\partial d_{i}}=0,\\ \overset{n}{\underset{i=1}{\sum }}d_{i}=d-nd_{\text{local}} \end{array}$$

by taking partial derivatives on (A.1) with respect to *d*_*i*_ and equating to 0. Solving (A.2) and each distance *d*_*i*_ can be determined as follows:

(A.3) di=(w4(1+a)E−b((4π)2/GtGrλ2)MlNf(n+1−i))−dlocal.

Since ∑_*i*=1_^*n*^ *d*_*i*_ = *d* − *nd*_local_, (A.3) can be derived as

(A.4) $$\begin{array}{c} d_{i}=\frac{d+nd_{\text{local}}}{\sum_{f=1}^{n}\left(1/\left(n+1-f\right)\right)\left(n+1-i\right)}-d_{\text{local}}. \end{array}$$
